# Risk of cancer in patients with dermatomyositis or polymyositis, and follow-up implications: a Scottish population-based cohort study

**DOI:** 10.1054/bjoc.2001.1699

**Published:** 2001-07

**Authors:** D Stockton, V R Doherty, D H Brewster

**Affiliations:** Scottish Cancer Intelligence Unit, Information & Statistics Division, Trinity Park House, Edinburgh, EH5 3SQ; Department of Dermatology, Royal Infirmary of Edinburgh, Lauriston Place, Edinburgh, EH3 9YW

**Keywords:** cancer, dermatomyositis, polymyositis, record linkage

## Abstract

We conducted a national, retrospective population-based cohort study of 705 patients hospitalized with a first diagnosis of dermatomyositis (DM) or polymyositis (PM) during 1982–1996 based on linkage of hospital discharge, cancer registration, and death records in Scotland. Risks of cancer were assessed by calculating standardized incidence ratios (SIR). A first malignancy was diagnosed concurrently or subsequently in 50 patients with DM (SIR 7.7, 95% CI 5.7–10.1), and 40 patients with PM (2.1, 1.5–2.9). Significantly elevated risks were observed for lung, cervix uteri, and ovarian cancer in patients with DM, and for Hodgkin’s disease in patients with PM. The excess risk of cancer was highest around the time of diagnosis, and for patients with DM remained high for at least 2 years. Risks were elevated for both sexes but only significantly so for females, and were highest in patients aged 45–74 years at the time of diagnosis for DM and 15–44 for PM. © 2001 Cancer Research Campaign http://www.bjcancer.com


British Journal of Cancer (2001) 85(1), 41–45
© 2001 Cancer Research Campaign
DOI: 10.1054/ bjoc.2001.1699, available online at http://www.idealibrary.com on IDEAL®

http://www.bjcancer.com

### Risk of cancer in patients with dermatomyositis or polymyositis, and follow-up implications: a Scottish population-based cohort study

**D Stockton[1], VR Doherty[2] and DH Brewster[1]**

[1]Scottish Cancer Intelligence Unit, Information & Statistics Division, Trinity Park House, Edinburgh EH5 3SQ; [2]Department of Dermatology, Royal Infirmary of Edinburgh, Lauriston Place, Edinburgh EH3 9YW

**Summary** We conducted a national, retrospective population-based cohort study of 705 patients hospitalized with a first diagnosis of dermatomyositis (DM) or polymyositis (PM) during 1982–1996 based on linkage of hospital discharge, cancer registration, and death records in Scotland. Risks of cancer were assessed by calculating standardized incidence ratios (SIR). A first malignancy was diagnosed concurrently or subsequently in 50 patients with DM (SIR 7.7, 95% CI 5.7–10.1), and 40 patients with PM (2.1, 1.5–2.9). Significantly elevated risks were observed for lung, cervix uteri, and ovarian cancer in patients with DM, and for Hodgkin's disease in patients with PM. The excess risk of cancer was highest around the time of diagnosis, and for patients with DM remained high for at least 2 years. Risks were elevated for both sexes but only significantly so for females, and were highest in patients aged 45–74 years at the time of diagnosis for DM and 15–44 for PM.
© 2001 Cancer Research Campaign http://www.bjcancer.com

**Keywords**: cancer; dermatomyositis; polymyositis; record linkage

An increased incidence of cancer in patients with dermatomyositis (DM) (Hidano et al, 1992; Sigurgeirsson et al, 1992; Rose et al, 1994; Selvaag et al, 1994; Zantos et al, 1994; Airio et al, 1995; Chow et al, 1995; Maos et al, 1998) and polymyositis (PM) (Sigurgeirsson et al, 1992; Zantos et al, 1994; Chow et al, 1995; Maos et al, 1998) has been reported in many studies, though with wide variations in the estimated risks. A few studies did not find an excess risk of cancer (Lakhanpal et al, 1986; Manchul et al, 1998) although these were hospital-based case–control studies of limited size. The increased risk has been reported to be greater in patients with DM and particularly among older (> 50 years) patients (Airio et al, 1995), and to decrease with time since the first DM or PM diagnosis (Chow et al, 1995). The present study was designed to estimate the risk of cancer among a population-based cohort of patients with DM or PM in Scotland, in relation to age at, and time since diagnosis, and to assess the implications for follow-up of patients with DM or PM.

### MATERIALS AND METHODS

### Patients

The Information and Statistics Division (ISD) of the National Health Service in Scotland has linked (at the time of analysis), using probability matching, information on all hospital inpatient episodes occurring between 1 January 1981 and 31 January 1998 in Scotland to death records for the same period and cancer registry records for patients diagnosed with cancer (ICD-9: 140–208) between 1980 and 1996 (Kendrick and Clarke, 1993).

*Received 19 September 2000*
*Accepted 2 April 2001*

*Correspondence to:* DH Brewster

For this study all records for patients with an initial diagnosis of DM (ICD-9: 710.3) or PM (ICD-9: 710.4) between 1982 and 1996 were selected from the linked database, giving a population-based dataset covering 15 years. Follow-up was until the end of 1996 because, at the time of analysis, cancer registration records were not complete beyond this date. The starting date was restricted to 1982 to reduce the erroneous inclusion of cases who had an earlier diagnosis of DM or PM. Additionally, the selected patients with DM or PM were probability matched to the cancer registry database from 1968 to 1979 to obtain information on all antecedent cancers in these patients. For patients with a diagnosis of both DM and PM ($n$ = 32), only the earliest diagnosis was included. Non-melanoma skin cancers were excluded as data are likely to be less complete for these cancers because they are relatively common and usually non-fatal, and do not usually involve hospital admission.

### Statistical analysis

Persons-years at risk were calculated from the date of admission to hospital with a first diagnosis of DM or PM, to either the date of death or 31 December 1996, whichever occurred first. The observed numbers of cancer cases were compared with those expected based on sex-, age- (five year age band to 85+) and period- (1982–86, 1987–91, 1992–96) specific incidence rates for Scotland. No attempt was made to adjust for socio-economic status as DM or PM showed no evidence of a socio-economic gradient (data not shown but available on request). The Scottish cancer incidence rates were calculated from (1) all incident cases and (2) only first malignancies. The standardized incidence ratio (SIR) was defined as the ratio of the observed to expected number of cases and the 95% confidence interval was estimated assuming that the observed number of cases followed a Poisson distribution.

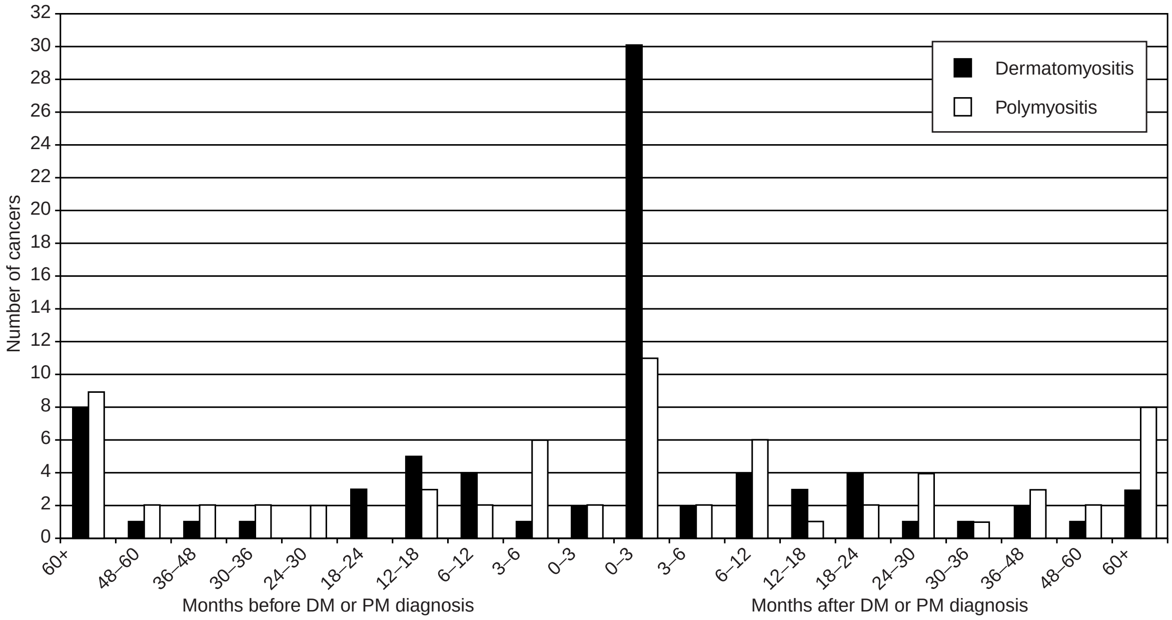

**Figure 1** Occurrence of cancer (only first primary malignancies are included) around DM or PM diagnosis

Two main analyses were performed:

(1) For estimation of the overall risk of cancer following DM or PM diagnosis – all patients with a malignancy diagnosed concurrently or after the first DM or PM diagnosis were included even if they had a previous cancer diagnosis; observed numbers were compared to those expected based on all incident cases in Scotland. Only the first cancer occurring concurrently or after the first DM or PM diagnosis was included.

(2) For estimation of the risk of cancer in patients with no sign of cancer at DM or PM diagnosis – all patients with an antecedent cancer or cancer diagnosed within 3 months of the first DM or PM diagnosis were excluded. The remaining observed cases were compared to expected numbers based on the incidence of first malignancies in Scotland; this was also done in relation to age at, and time since, diagnosis of DM or PM. Only the first cancer occurring after the first DM or PM diagnosis was included.

### RESULTS

Overall, 705 patients with an initial diagnosis of DM ($n$ = 286) or PM (419) between January 1982 and December 1996 were identified. There were 272 men and 433 women, of whom 44 (6%) were aged under 15 years, 167 (24%) were between the ages of 15 and 44 years, 414 (59%) were aged 45 to 74, and 80 (11%) were aged 75 years or older. The cancer history of these patients can be seen in Figure 1, with the incidence of cancer being distributed evenly both before and after the diagnosis of DM or PM. A large number of cancers were diagnosed at the same time or within 3 months of the DM or PM diagnosis. The numbers reduced in both directions as time from DM or PM diagnosis increased.

### Dermatomyositis

Among patients with a first diagnosis of DM (97 men and 189 women), cancer was (or had previously been) diagnosed in 77 (27%) patients.

**Table 1** Observed (Obs) numbers of cancers and standardized incidence ratios (SIR) among patients with DM or PM diagnosed in Scotland 1982–1996

| Type | Cancer inclusion criteria | Time since DM/PM diagnosis | Overall | | | Males | | | Females | | |
|---|---|---|---|---|---|---|---|---|---|---|---|
| | | | Obs | SIR | 95% CI | Obs | SIR | 95% CI | Obs | SIR | 95% CI |
| Dermatomyositis | First cancer[a] | Concurrently or after[c] | 50 | 7.7 | (5.7, 10.1) | 20 | 9.2 | (5.6, 14.1) | 30 | 6.9 | (4.7, 9.9) |
| | | After[d] | 20 | 3.3 | (2.0, 5.1) | 5 | 2.5 | (0.8, 5.9) | 15 | 3.7 | (2.1, 6.1) |
| | Any cancer[b] | Concurrently or after[c] | 51 | 5.8 | (4.4, 7.7) | 20 | 7.4 | (4.5, 11.4) | 31 | 5.2 | (3.5, 7.3) |
| | | After[d] | 22 | 2.7 | (1.7, 4.1) | 5 | 2.0 | (0.7, 4.8) | 17 | 3.0 | (1.7, 4.8) |
| Polymyositis | First cancer[a] | Concurrently or after[c] | 40 | 2.1 | (1.5, 2.9) | 12 | 1.3 | (0.7, 2.2) | 28 | 3.0 | (2.0, 4.4) |
| | | After[d] | 29 | 1.6 | (1.1, 2.4) | 8 | 0.9 | (0.4, 1.8) | 21 | 2.4 | (1.5, 3.7) |
| | Any cancer[b] | Concurrently or after[c] | 43 | 1.8 | (1.3, 2.4) | 13 | 1.1 | (0.6, 1.9) | 30 | 2.5 | (1.7, 3.5) |
| | | After[d] | 32 | 1.4 | (1.0, 2.0) | 9 | 0.8 | (0.4, 1.5) | 23 | 2.0 | (1.3, 3.0) |

[a]Only first primary malignancies are included in the calculation of both the observed and expected. [b]Including second cancers in patients who had a cancer prior to the DM or PM diagnosis. [c]Cancers diagnosed at the same time or after the DM or PM diagnosis. [d]Cancers diagnosed at least 3 months after the DM or PM diagnosis.

 © 2001 Cancer Research Campaign

**Table 2** Observed (Obs) number of cancers and standardized incidence ratios (SIR) of cancers diagnosed among patients with DM or PM in Scotland 1982–1996[a]

| Type | ICD9 code | Cancer | Concurrently or after[b] Obs | SIR | 95% CI | After[c] Obs | SIR | 95% CI |
|---|---|---|---|---|---|---|---|---|
| DM | 150 | Oesophagus | 1 | 6.2 | (0.2, 34.4) | 0 | | |
| | 151 | Stomach | 3 | 10.0 | (2.1, 29.2) | 0 | | |
| | 153 | Colon | 7 | 12.8 | (5.1, 26.3) | 1 | 2.0 | (0, 10.9) |
| | 162 | Lung | 19 | 15.6 | (9.4, 24.3) | 6 | 5.3 | (2, 11.6) |
| | 174 | Breast (women) | 3 | 2.7 | (0.6, 7.9) | 3 | 2.8 | (0.6, 8.3) |
| | 180 | Cervix uteri | 2 | 11.4 | (1.4, 41.1) | 2 | 11.9 | (1.4, 43) |
| | 183 | Ovary | 2 | 9.1 | (1.1, 32.7) | 2 | 9.6 | (1.2, 34.5) |
| | 185 | Prostate | 1 | 3.7 | (0.1, 20.8) | 1 | 4.2 | (0.1, 23.1) |
| | 188 | Bladder | 1 | 3.2 | (0.1, 18.1) | 0 | | |
| | 196–199 | Secondary and unspecified malignancy | 6 | 18.5 | (6.8, 40.4) | 3 | 10.0 | (2.1, 29.1) |
| | 201 | Hodgkin's disease | 1 | 28.4 | (0.7, 157.9) | 1 | 29.8 | (0.8, 165.9) |
| | 202 | Non-Hodgkin's lymphoma | 2 | 13.3 | (1.6, 48) | 1 | 7.1 | (0.2, 39.8) |
| PM | 140 | Lip | 1 | 17.1 | (0.4, 95.1) | 1 | 17.8 | (0.4, 99) |
| | 141 | Tongue | 1 | 15.4 | (0.4, 85.9) | 1 | 16.2 | (0.4, 90.4) |
| | 143 | Gum | 1 | 71.5 | (1.8, 398.3) | 1 | 75.2 | (1.9, 418.7) |
| | 153 | Colon | 1 | 0.6 | (0, 3.4) | 1 | 0.6 | (0, 3.6) |
| | 154 | Rectum | 2 | 2.4 | (0.3, 8.7) | 2 | 2.5 | (0.3, 9.1) |
| | 155 | Liver | 1 | 6.7 | (0.2, 37.3) | 0 | | |
| | 156 | Gall bladder | 1 | 8.4 | (0.2, 47) | 1 | 8.8 | (0.2, 49.1) |
| | 162 | Lung | 8 | 1.9 | (0.9, 3.8) | 5 | 1.3 | (0.4, 3) |
| | 172 | Malignant melanoma of skin | 1 | 3.5 | (0.1, 19.4) | 1 | 3.7 | (0.1, 20.4) |
| | 174 | Breast (women) | 5 | 2.3 | (0.7, 5.4) | 5 | 2.4 | (0.8, 5.6) |
| | 182 | Corpus uteri | 1 | 3.6 | (0.1, 20) | 1 | 3.8 | (0.1, 21) |
| | 183 | Ovary | 1 | 2.3 | (0.1, 12.6) | 1 | 2.4 | (0.1, 13.2) |
| | 185 | Prostate | 4 | 3.2 | (0.9, 8.3) | 2 | 1.7 | (0.2, 6.2) |
| | 189 | Kidney | 1 | 2.7 | (0.1, 15.2) | 0 | | |
| | 191 | Brain | 1 | 4.9 | (0.1, 27.5) | 0 | | |
| | 193 | Thyroid | 1 | 15.2 | (0.4, 84.7) | 0 | | |
| | 196–199 | Secondary and unspecified malignancy | 3 | 3.1 | (0.6, 9) | 3 | 3.2 | (0.7, 9.4) |
| | 201 | Hodgkin's disease | 2 | 31.0 | (3.8, 112) | 2 | 32.5 | (3.9, 117.2) |
| | 202 | Non-Hodgkin's lymphoma | 2 | 5.0 | (0.6, 18.1) | 1 | 2.6 | (0.1, 14.7) |
| | 203 | Multiple myeloma | 2 | 8.6 | (1, 31.2) | 1 | 4.5 | (0.1, 25.2) |

[a]Only first primary malignancies are included in the calculation of both the observed and expected. [b]Cancers diagnosed at the same time or after the DM or PM diagnosis. [c]Cancers diagnosed at least 3 months after the DM or PM diagnosis.

A first malignancy was diagnosed concurrently or after the DM diagnosis in 50 patients giving a standardized incidence ratio (SIR) of 7.7 (95% confidence interval 5.7–10.1) compared to that expected based on the incidence of first malignancies in Scotland as a whole. When cancers diagnosed in the first 3 months following diagnosis were excluded (under the assumption that they are concurrent diagnoses made while the patient was under heightened surveillance) there was a smaller, but still significant, 3-fold increased risk with an SIR of 3.3 (2.0–5.1) (Table 1). The increase is seen in both men and women. When individual cancers were analysed, a significant increase was seen for a number of cancers, in particular, a 5-fold increased risk for lung, 12-fold for cervical, and 10-fold for ovarian cancer (Table 2).

For all first malignancies combined there was a very high increased risk (SIR 65.6) in the first 3 months after diagnosis, elevated risks which were bordering on or statistically significant for the following periods up to 2 years, and then elevated but non-significant risks thereafter (Figure 2). The increased risks were significant in the 45–74 age group, with SIRs of 4.8 (95% CI 0.6–17.4), 3.6 (2.0–5.9) and 2.1 (0.4–6.1) in the age groups 15–44, 45–74 and 75+, respectively. No cancers were seen in the 35 children (aged < 15) recorded as having DM.

### Polymyositis

Among patients with a first diagnosis of PM (175 men and 244 women), cancer was (or had previously been) diagnosed in 71 (17%) patients.

A first malignancy was diagnosed concurrently or after the PM diagnosis in 40 patients (SIR 2.1, 1.5–2.9). When cancers diagnosed in the first 3 months following diagnosis were excluded the SIR was still significantly high (SIR 1.6, 1.1–2.4) (Table 1). The increase was mainly seen in women with a non-significant excess for men which disappeared when the cancers diagnosed within 3 months of PM are excluded. The number of observed cancers were small when individual cancers were analysed, however there was a significant increase in risk for Hodgkin's disease (SIR 32.5, 3.9–117.2) (Table 2).

For all first malignancies combined there was a high increased risk (SIR 12.0) in the first 3 months after diagnosis; subsequently the risk reduced leaving a small, mainly non-significant excess thereafter (Figure 2). The increased risks were significant in the 15–44 age group, with SIRs of 8.9 (2.4–22.8), 1.5 (0.9–2.4) and 1.3 (0.5–2.6) in the age groups 15–44, 45–74 and 75+, respectively. No cancers were seen in the 9 children recorded as having PM.

© 2001 Cancer Research Campaign

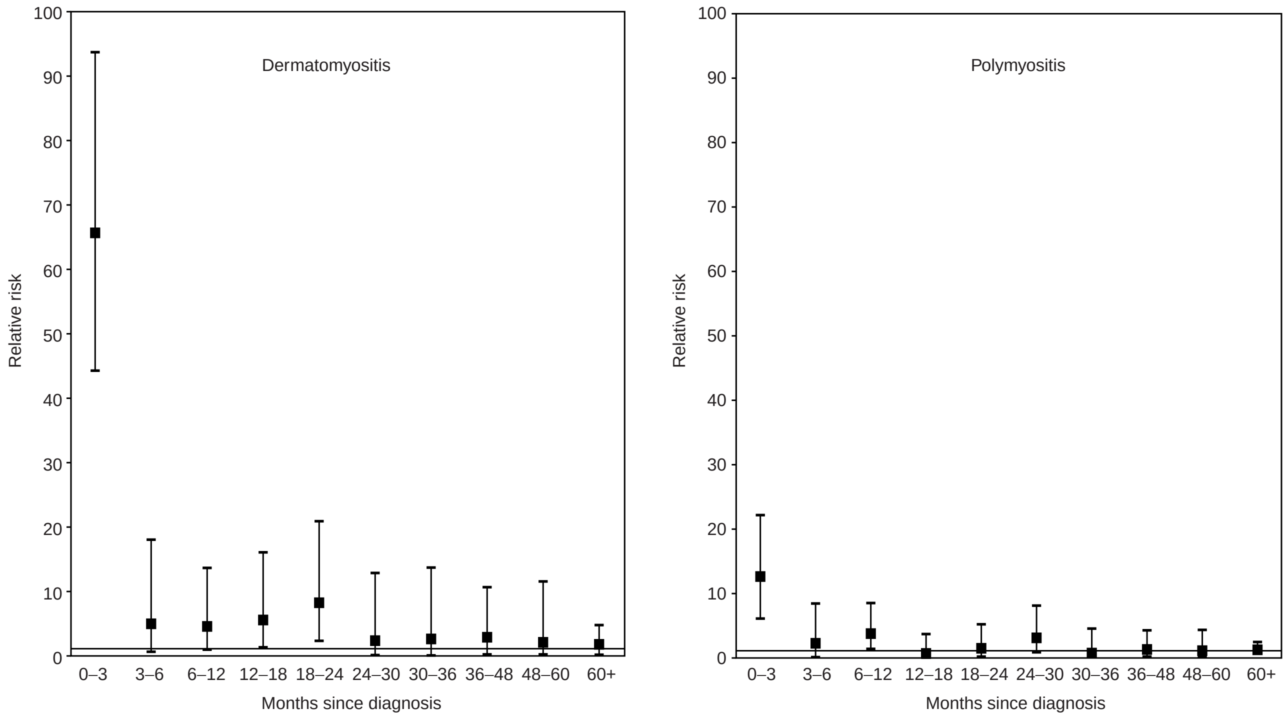

**Figure 2** Temporal trends in risk of cancer (only first primary malignancies are included in the calculation of both the observed and expected) after DM or PM diagnosis

### DISCUSSION

We evaluated the association between DM and PM and a subsequent cancer in a large population-based cohort and confirmed an increased risk of several types of cancer. In particular, there was a strong association between DM and lung, cervical, and ovarian cancers; and between PM and Hodgkin's disease.

It should be noted that, although some of the risks found are large, they are based on small numbers of excess cases, and are the product of multiple tests of statistical significance. Nevertheless, other population-based studies have also found an increased risk for cancers of the lung, female genital organs and ovary among patients with DM (Sigurgeirsson et al, 1992; Airio et al, 1995; Chow et al, 1995). The results for PM and cancer are not as well supported: one study suggests that patients may be prone to certain tumours, such as lymphoma, myeloma or leukaemia due to a compromised immune state, or as a result of immuno-suppressive or cytotoxic therapy (Chow et al, 1995). However, another study failed to find a cancer excess among DM patients treated with cytotoxic drugs (Airio et al, 1995).

It is likely that some of the excess cancer occurrence reported among patients with DM and PM reflects intensive investigation due to the known association between DM or PM and cancer (Lakhanpal et al, 1986; Manchul et al, 1998). Its effects are probably most evident around the time of diagnosis of DM or PM (Figure 1). As an attempt to account for this surveillance bias, risk of cancer was studied, excluding those diagnosed within 3 months of the DM or PM diagnosis, and we found a 3-fold increased risk of cancer for DM patients and a 1.6-fold increased risk for PM patients (Table 1).

In line with a similar study from Denmark (Chow et al, 1995), we restricted our analysis to first cancers arising after the (first) diagnosis of DM or PM. This avoids the confounding effect of cancer itself being a risk factor for subsequent cancers, through genetic susceptibility, shared risk factors, or the effects of therapy. However, it means that our estimates of risk may be conservative.

Some patients may have been included erroneously as having a first diagnosis of DM or PM when they actually had a previous diagnosis prior to 1981. This would have the effect of over-estimating the risk of cancer due to under-estimation of the person-years of observation. From the initial data set of 769 cases diagnosed over the period 1981–1996, 31 patients had 2 admissions for DM and/or PM (15 within one year, 11 within 1–5 years, and 5 greater than 5 years apart). From our analysis, restricted to DM and PM patients diagnosed from 1982 onwards, we estimate that not more than 10 (1%) patients will have been wrongly included as first diagnoses.

The results of our study relate only to patients with DM or PM who are hospitalized at some stage of their disease, although the diagnosis of DM or PM is likely to be more secure in such patients. The risk of cancer will probably be either similar or lower in patients who are not hospitalized. If any bias exists, therefore, the effect will have been to over-estimate the risk of cancer with respect to *all* patients with DM or PM.

Although there is limited published evidence about the quality of hospital discharge data in Scotland, a recent study estimated that, during the early to mid 1990s, the overall accuracy of coding diagnosis approached 90% (Harley and Jones, 1996). The quality of cancer registry data in Scotland is also believed to be high, both in terms of accuracy (Brewster et al, 1994) and ascertainment (Brewster et al, 1997). The method of record linkage used in Scotland is estimated to result in mismatched records in less than 2% of cases (Kendrick and Clarke, 1993). Although we are unable to quantify losses to follow-up through migration, they are unlikely to represent a major source of bias among this group of patients and would result in an under-estimation of risk.

© 2001 Cancer Research Campaign

In a similar study in Sweden (Sigurgeirsson et al, 1992) the validity of hospital diagnoses of DM and PM was reviewed in a 9% sample of hospital medical records. Only 7% of this sample were judged probably not to have either condition. In contrast, another similar study in Finland (Airio et al, 1995) assessed the validity of DM and PM diagnoses in hospital episode records by checking manually all available medical records, and found that 33% of their cases were misclassified. In our study, it was not feasible to check the DM and PM diagnoses manually. We found that 11% of the patients in our study diagnosed during 1982–90 had a subsequent in-patient diagnosis of conditions, such as polymyalgia rheumatica, which the Finnish study identified as leading to misclassification. (We restricted our cohort to DM and PM patients diagnosed in the years stated to allow time for subsequent diagnoses to occur.) The effect of any diagnostic misclassification in relation to DM or PM would be to decrease our estimates of excess risk.

In conclusion, we have estimated the long-term risk of cancer among a population-based cohort of patients with DM or PM. We found an increased risk of cancer following the diagnosis of DM and PM, which was significantly raised for females, and for DM patients aged between 45 and 75 years, and PM patients aged 15 to 44 years. The elevated risk of cancer was seen within the first 3 months for patients with PM and the first 2 years for patients with DM. A high index of suspicion for malignancy is justified for patients with DM (approximately 20 new cases per annum in Scotland), particularly during the first 2 years after diagnosis, and to a lesser extent PM (approximately 28 new cases per annum in Scotland), in the first year following diagnosis.

### ACKNOWLEDGEMENTS

The contribution of everyone involved in the collection of routine health-related data in Scotland is gratefully acknowledged. We are also grateful for the input of the Record Linkage Team in the Information and Statistics Division of the National Health Service in Scotland.

### REFERENCES

Airio A, Pukkala E and Isomäki H (1995) Elevated cancer incidence in patients with dermatomyositis: a population based study. *J Rheumatol* **22**: 1300–1303

Brewster D, Crichton J and Muir C (1994) How accurate are Scottish cancer registration data? *Br J Cancer* **70**: 954–959

Brewster D, Crichton J, Harvey JC and Dawson G (1997) Completeness of case ascertainment in a Scottish Regional Cancer Registry. *Public Health* **111**: 339–343

Chow WH, Gridley G, Mellemkjær L, McLaughlin JK, Olsen JH and Fraumeni JF Jr (1995) Cancer risk following polymyositis and dermatomyositis: a nationwide cohort study in Denmark. *Cancer Causes Control* **6**: 9–13

Harley K and Jones C (1996) Quality of Scottish Morbidity Record (SMR) data. *Health Bulletin (Edinburgh)* **54**: 410–417

Hidano A, Torikai S, Uemura T and Shimizu S (1992) Malignancy and interstitial pneumonitis as fatal complications in dermatomyositis. *J Dermatol* **19**: 153–160

Kendrick S and Clarke J (1993) The Scottish Record Linkage System. *Health Bulletin (Edinburgh)* **51**: 72–79

Lakhanpal S, Bunch TW, Ilstrup DM and Melton LJ (1986) Polymyositis-dermatomyositis and malignant lesions: does an association exist? *Mayo Clin Proc* **61**: 645–653

Manchul LA, Jin A, Pritchard KI, Tenenbaum J, Boyd NF, Lee P, Germanson T and Gordon DA (1998) The frequency of malignant neoplasms in patients with polymyositis-dermatomyositis. A controlled study. *Arch Intern Med* **145**: 1835–1839

Maos CR, Langevitz P, Livneh A, Blumstein Z, Sadeh M, Bank I, Gur H and Ehrenfeld M (1998) High incidence of malignancies in patients with dermatomyositis and polymyositis: an 11-year analysis. *Sem Arthrit Rheumat* **27**: 319–324

Rose C, Hatron PY, Brouillard M, Hachulla E, Gossett D, Marlier C, Piette F and Devulder B (1994) Predictive signs of cancers in dermatomyositis. Study of 29 cases. *Rev Med Intern* **15**: 19–24

Selvaag E, Thune P and Austad J (1994) Dermatomyositis and cancer. A retrospective study. *Tidsskrift Norke Laegeforening* **114**: 2378–2380

Sigurgeirsson B, Lindelöf B, Edhag O and Allander E (1992) Risk of cancer in patients with dermatomyositis or polymyositis. *New Engl J Med* **326**: 363–367

Zantos D, Zhang Y and Felson D (1994) The overall and temporal association of cancer with polymyositis and dermatomyositis. *J Rheumatol* **21**: 1855–1859

© 2001 Cancer Research Campaign
